# Predictors of and hospital-level variation in intensive care unit readmissions in Japan: a nationwide inpatient database study

**DOI:** 10.1186/s40560-025-00838-3

**Published:** 2025-11-29

**Authors:** Hiroyuki Ohbe, Yusuke Sasabuchi, Yuya Kimura, Hiroki Matsui, Kiyohide Fushimi, Hideo Yasunaga, Daisuke Kudo

**Affiliations:** 1 Department of Emergency and Critical Care Medicine, Tohoku University Hospital, 1-1 Seiryo-machi, Aoba-ku, Sendai, 980-8574 Japan; 2 Department of Clinical Epidemiology and Health Economics, School of Public Health, The University of Tokyo, 7-3-1 Hongo, Bunkyo-ku, Tokyo, 113-0033 Japan; 3 Department of Real-world Evidence, The Graduate School of Medicine, The University of Tokyo, 7-3-1 Hongo, Bunkyo-ku, Tokyo, 113-0033 Japan; 4 Department of Health Services Research, Graduate School of Medicine, The University of Tokyo, 7-3-1 Hongo, Bunkyo-ku, Tokyo, 113-0033 Japan; 5 Department of Health Policy and Informatics, Institute of Science Tokyo Graduate School, 2-12-1 Ookayama, Meguro-ku, Tokyo, 152-8550 Japan; 6 Division of Emergency and Critical Care Medicine, Tohoku University Graduate School of Medicine, 2-1 Seiryo-machi, Aoba-ku, Sendai, Miyagi 980-8575 Japan

**Keywords:** Intensive care unit, ICU readmission, Quality indicator, Hospital variation, Japan

## Abstract

**Background:**

Intensive care unit (ICU) readmission is a widely recognized marker of patient outcomes and organizational performance. Early ICU readmission—commonly defined as a return to the ICU within 48 h or 2 full calendar days—has been posited as a quality indicator. However, its appropriateness as a quality indicator remains debatable, and evidence from Japan is limited.

**Methods:**

We conducted a retrospective nationwide cohort study in Japan using data from the Diagnosis Procedure Combination database linked with the Hospital Bed Function Report from 2018 through 2023. Adults who were discharged alive after their initial ICU stay, with available Sequential Organ Failure Assessment (SOFA) scores at ICU admission and discharge were included. The primary outcome was early ICU readmission, defined as readmission to the ICU within ≤ 2 full calendar days after the initial ICU discharge. All ICU readmissions (early and late combined) during the same hospitalization were also assessed. Patient- and hospital-level predictors were evaluated using multilevel logistic regression. Between-hospital variability was quantified using intraclass correlation coefficients (ICC) and median odds ratios (MOR).

**Results:**

Of 552,545 eligible patients across 401 hospitals, 22,112 patients (4.0%) underwent ICU readmission; 4728 patients (0.9%) required early readmission. The temporal distribution lacked an early peak, with 32.8% of readmissions occurring ≥ 14 days and 12.2% occurring ≥ 30 days after the initial ICU discharge. Early readmission was associated with higher Charlson Comorbidity Index values, emergency surgery, and elevated SOFA scores at ICU discharge, particularly residual respiratory and circulatory dysfunction. SOFA scores at ICU admission and ICU stay ≥ 7 days were not predictive of early readmission. Intermediate care transfer conferred protection against early but not overall readmission. Hospital-level variation was substantial (ICC, 14–15%; MOR, approximately 2.0) and persisted after adjusting for patient- and hospital-level factors.

**Conclusions:**

In Japan, early ICU readmissions are less frequent than in most Western countries and often occur long after discharge. Discharge severity scores, rather than disease severity at admission, served as the key predictors of readmission. Persistent unexplained hospital-level variations suggest that the ICU readmission rate alone is insufficient as a standalone indicator of the quality of care.

**Supplementary Information:**

The online version contains supplementary material available at 10.1186/s40560-025-00838-3.

## Background

Intensive care unit (ICU) readmission during the same hospitalization is recognized internationally as a key marker of patient outcomes and organizational performance, owing to its association with longer hospital stays, higher costs, and increased mortality [1–4]. The European Society of Intensive Care Medicine, Society for Critical Care Medicine in the United States, and Australia and New Zealand Intensive Care Society have posited early ICU readmission—commonly defined as a patient’s return to the ICU within 48 h or 2 full calendar days of discharge—as a key quality indicator because it may signal premature or unsuccessful ICU discharge [1, 2, 5]. However, several investigators have questioned its validity as a quality indicator, noting a weak association between ICU readmission rates and patient outcomes after case-mix adjustment [6, 7], substantial inter-hospital variability in readmission rates [6, 8], and the inherent limitation that only patients who survive their initial ICU stay can be at risk for readmission [8].

Large multicenter cohort studies and meta-analyses from North America and Europe have reported overall ICU readmission rates of approximately 4–10%, while early readmissions, defined as return within 48 h or 2 full calendar days, account for 2–3% of ICU discharges, with significant inter-hospital variability [8–14]. Previous studies have identified multiple risk factors for ICU readmission, including advanced age, higher illness severity scores, emergency or medical admissions, need for invasive mechanical ventilation, and residual organ dysfunction at ICU discharge—particularly ongoing vasopressor use or respiratory support [8–11].

Despite these insights, evidence from Japan, a country with relatively limited ICU capacity (5.8 ICU beds per 100,000 population in 2022), remains scarce. Most previous Japanese studies were single-center analyses [15]. A recent study of 57 ICUs (approximately 20% of all Japanese ICUs) conducted using the Japanese Intensive Care Patient Database (JIPAD) reported an overall readmission rate of 4.3% [16]. That study identified higher Acute Physiology and Chronic Health Evaluation (APACHE) III scores at ICU admission, renal replacement therapy, noninvasive positive pressure ventilation, intra-aortic balloon pumping, and longer ICU stay as risk factors for readmission [16]. However, the JIPAD only covers approximately 20% of all Japanese ICUs and does not assess risk at the point of ICU discharge. Moreover, no prior study has used a national inpatient database to quantify the ICU readmission risk or between-hospital variability across a broad spectrum of Japanese hospitals. Consequently, it is unclear whether Japan’s readmission patterns align with international benchmarks, or whether institutional factors contribute significantly to the observed variation.

To address these gaps in evidence, we conducted a nationwide cohort study using the Diagnosis Procedure Combination (DPC) database linked to the Hospital Bed Function Report. Our objectives were to (1) describe the early and overall ICU readmission rates in Japan, (2) identify the patient- and hospital-level predictors of readmission, and (3) quantify hospital-level variations in the ICU readmission risk.

## Methods

### Data source

We conducted this retrospective, nationwide cohort study using data extracted from the Diagnosis Procedure Combination (DPC) database and the Hospital Bed Function Report in Japan [17, 18]. The DPC database encompasses administrative claims data and discharge summaries from more than 1500 voluntarily participating acute-care hospitals, covering nearly 70% of acute-care beds in Japan [17]. It is a repository of detailed patient-level information, such as demographics, diagnoses recorded using the International Classification of Diseases, Tenth Revision (ICD-10) codes, daily procedures, medications, reimbursements, and discharge status. The database possesses high validity for general procedures (> 90% sensitivity and specificity) and moderate validity for diagnoses (sensitivity, 78.9%; specificity, 93.2%) [19].

We also used the Hospital Bed Function Report, published annually by the Ministry of Health, Labour, and Welfare of Japan, which provides hospital-level characteristics as of July 1 annually [18]. The data include hospital type, ward type, number of beds, and annual patient volume. These data are linked to the DPC database using hospital identifiers and fiscal years.

This study was approved by the Institutional Review Board of the University of Tokyo (approval number: 3501-5; approval date: May 19, 2021). The requirement for informed consent was waived as all data were de-identified. The study was conducted in accordance with the tenets of the Declaration of Helsinki.

### Definitions of the ICU

Under Japan’s national health insurance system, ICUs are classified under medical reimbursement codes A3011–A3014. Codes A3011 and A3012 require the presence of at least two on-site intensivists 24 h a day, 7 days a week, as well as a dedicated ICU nurse, defined as a full-time nurse with at least 5 years’ critical care experience and specialized training who is assigned to the ICU for at least 20 h per week, and a clinical engineer who is present in the hospital at all times. A3012 is additionally authorized to treat severe burns. Codes A3013 and A3014 require the presence of at least one non-intensivist physician on site 24 h a day but do not mandate the presence of a dedicated ICU nurse or clinical engineer; A3014 is also authorized to treat severe burns. ICUs under all four codes must maintain round-the-clock nursing with a nurse-to-patient ratio exceeding 1:2, employ physicians dedicated to ICU coverage during night shifts, and possess the equipment necessary to care for critically ill patients. Neonatal and obstetric ICUs were not included. Although emergency and critical care units (A3002 and A3004) are also recognized as ICUs in Japan, they were not included in this study because recording the Sequential Organ Failure Assessment (SOFA) score in the DPC database is not mandatory for patients admitted to these units, as described later. Additional details are provided in Supplementary Table 1.

Since April 1, 2018 (A3011 and A3012) and October 1, 2020 (A3013 and A3014), healthcare providers in Japan have to compulsorily record the SOFA scores along with the corresponding recording dates at ICU admission and discharge in “Format 1” of the DPC database to qualify for reimbursement. The SOFA score assesses the performance of six organ systems, and each system is assigned a point value from 0 (normal) to 4 (high degree of dysfunction/failure). The total SOFA score ranges from 0 to 24 [20]. For each ICU admission and discharge day, the database stores the six organ-specific SOFA subscores measured at the time of occurrence of the “worst” total SOFA score.

### Study population

We included patients admitted to the ICU for whom the SOFA scores at ICU admission and discharge were available in the DPC database between April 1, 2018, and March 31, 2023. We excluded patients if (1) their hospital data could not be linked between the DPC database and the Hospital Bed Function Report 2018–2022, (2) the hospital’s annual ICU admission volume was fewer than 50 cases in a given year, or (3) any SOFA subscore at ICU admission or discharge was missing. In addition, consistent with previous studies [9], (4) patients who died during the initial ICU admission or (5) patients who survived the initial ICU stay but subsequently died in the hospital without ICU readmission were excluded.

### Outcomes and variables

The primary outcome was early ICU readmission, defined as readmission to the ICU within ≤ 2 full calendar days after the initial ICU discharge. This definition was selected because early ICU readmission is recommended by academic societies as a qualitative indicator of ICU performance and is reportedly a marker of premature or unsuccessful ICU discharge [1, 2, 5], whereas late ICU readmission may reflect new or unrelated clinical deterioration [9]. All ICU readmissions (early and late combined) during the same hospitalization were also evaluated. The secondary outcomes included in-hospital mortality, ICU mortality during the second ICU stay, respective lengths of ICU and hospital stay, and total hospitalization costs. ICU readmission and lengths of ICU stay were identified based on “Format 1” of the DPC database, which includes mandatory SOFA recording fields for ICU admission and discharge. These fields enable identification of both initial and subsequent ICU stays, irrespective of the 14-day reimbursement limit.

The patient-level variables included age, sex, Charlson Comorbidity Index, long-term care needs, admission classification, primary diagnosis category, total SOFA score at ICU admission, receipt of organ support therapy at ICU discharge, total SOFA score at ICU discharge, SOFA subscore ≥ 2 at ICU discharge, length of first ICU stay, step-down transfer from the ICU to the intermediate care unit (IMCU), fiscal year (2018–2019 and 2020–2022), season (spring: March–May, summer: June–August, fall: September–November, and winter: December–February), and weekend discharge from the ICU (defined as Saturdays and Sundays but excluding national holidays). Hospital-level variables included the reimbursement code of the ICU, number of ICU beds, hospital with IMCU beds, total number of hospital beds, academic hospital, annual volume of ambulance acceptance, and tertiary emergency hospital. Additionally, we collated data on the receipt of life-sustaining therapy during ICU stay, on the day of ICU discharge, and for ≥ 2 consecutive days from the day of ICU discharge. Life-sustaining therapies included invasive mechanical ventilation, vasopressors (noradrenaline or adrenaline), cardiopulmonary resuscitation, mechanical circularity support, and renal replacement therapies. No data were missing for the variables included in the final analysis.

### Statistical analysis

Three main analyses were conducted. First, we used standardized mean differences (SMD) to compare the characteristics of patients with and without ICU readmission; an absolute SMD ≤ 10% denoted negligible imbalance between the groups [21]. We also constructed a histogram to illustrate the distribution of time from the initial ICU discharge to the first ICU readmission.

Second, to identify the risk factors for ICU readmission, we performed multilevel logistic regression using the predictors available up to the time of initial ICU discharge, including all the patient- and hospital-level variables listed in Table 1. The models included random intercepts for hospital to account for clustering, and adjusted odds ratios (ORs) with 95% confidence intervals (CIs) were reported. Separate models were fitted for early and all ICU readmissions. We additionally performed a time-to-event sensitivity analysis using a cause-specific Cox proportional hazards model, treating in-hospital death as a censoring event. This sensitivity analysis included patients who died in-hospital without ICU readmission (previously excluded from the main analysis) and accounted for within-hospital clustering using cluster-robust standard errors.

**Table 1:** Patient and hospital characteristics by ICU readmission status

Characteristics	Patients discharged alive without ICU readmission n = 530,433	Patients with early ICU readmission n = 4728	SMD % (non-readmission vs. early readmission)	All patients with ICU readmission n = 22,112	SMD % (non-readmission vs. all readmission)
Patient characteristics					
Age, years	68.5 (14.9)	69.6 (14.2)	8	69.5 (14.5)	7
Male	322,508 (60.8)	3,076 (65.1)	9	14,583 (66.0)	11
Charlson comorbidity index	1.2 (1.5)	1.5 (1.6)	16	1.5 (1.6)	17
Long-term care needs					
No care needs	480,723 (90.6)	4,169 (88.2)	− 8	19,295 (87.3)	− 11
SL1-2 and CNL1	26,500 (5.0)	293 (6.2)	5	1,487 (6.7)	7
CNL 2–5	23,210 (4.4)	266 (5.6)	6	1,330 (6.0)	7
Admission classification					
Elective surgery	289,516 (54.6)	1,835 (38.8)	− 32	7,696 (34.8)	− 41
Emergency surgery	87,668 (16.5)	1,411 (29.8)	32	8,609 (38.9)	52
Non-surgery	153,249 (28.9)	1,482 (31.3)	5	5,807 (26.3)	− 6
Primary diagnosis category					
Cancer	155,447 (29.3)	996 (21.1)	− 19	4,743 (21.4)	− 18
Acute coronary syndrome	58,712 (11.1)	580 (12.3)	4	2,460 (11.1)	0
Aortic dissection or aneurysm	62,200 (11.7)	755 (16.0)	12	3,882 (17.6)	17
Stroke	37,706 (7.1)	276 (5.8)	− 5	1,518 (6.9)	− 1
Acute abdominal diseases	38,107 (7.2)	530 (11.2)	14	2,565 (11.6)	15
Acute heart failure	26,770 (5.0)	366 (7.7)	11	1,898 (8.6)	14
Sepsis	17,810 (3.4)	349 (7.4)	18	1,741 (7.9)	20
Trauma	15,274 (2.9)	178 (3.8)	5	906 (4.1)	7
Total SOFA score at ICU admission	3.0 (1.0–6.0)	5.0 (2.0–8.0)	48	5.0 (2.0–8.0)	42
Total SOFA score at ICU discharge	2.0 (1.0–4.0)	4.0 (2.0–6.0)	61	3.0 (2.0–5.0)	47
SOFA subscore ≥ 2 at ICU discharge					
Respiratory	112,166 (21.1)	1,735 (36.7)	35	6,887 (31.1)	23
Platelet	68,139 (12.8)	1,035 (21.9)	24	4,152 (18.8)	16
Liver	29,253 (5.5)	490 (10.4)	18	2,177 (9.8)	16
Circulatory	37,288 (7.0)	865 (18.3)	34	2,804 (12.7)	19
Neurological	38,983 (7.3)	681 (14.4)	23	3,029 (13.7)	21
Renal	48,925 (9.2)	908 (19.2)	29	4,232 (19.1)	29
Length of first ICU stay					
1 day	39,114 (7.4)	265 (5.6)	− 7	1,414 (6.4)	− 4
2 days	267,650 (50.5)	1,729 (36.6)	− 28	6,969 (31.5)	− 39
3–6 days	165,258 (31.2)	1,771 (37.5)	13	8,196 (37.1)	12
≥ 7 days	58,411 (11.0)	963 (20.4)	26	5,533 (25.0)	37
Step-down transfer from ICU to IMCU	51,371 (9.7)	528 (11.2)	5	2,798 (12.7)	9
Fiscal year					
2018–2019	141,951 (26.8)	1,316 (27.8)	2	6,356 (28.7)	4
2020–2022	388,482 (73.2)	3,412 (72.2)	− 2	15,756 (71.3)	− 4
Season					
Spring	124,322 (23.4)	1,066 (22.5)	− 2	5,185 (23.4)	0
Summer	130,407 (24.6)	1,213 (25.7)	2	5,524 (25.0)	1
Fall	138,637 (26.1)	1,288 (27.2)	2	5,969 (27.0)	2
Winter	137,067 (25.8)	1,161 (24.6)	− 3	5,434 (24.6)	− 3
Weekend discharge from ICU	93,218 (17.6)	775 (16.4)	− 3	3,503 (15.8)	− 5
Hospital characteristics					
Reimbursement under ICU code					
A3011	226,302 (42.7)	2,398 (50.7)	16	10,452 (47.3)	9
A3012	120,359 (22.7)	1,114 (23.6)	2	5,376 (24.3)	4
A3013	139,113 (26.2)	920 (19.5)	− 16	4,673 (21.1)	− 12
A3014	44,659 (8.4)	296 (6.3)	− 8	1,611 (7.3)	− 4
Number of ICU beds	14 (8–22)	16 (10–23)	17	16 (10–22)	9
Hospital with IMCU beds	429,374 (80.9)	3,868 (81.8)	2	18,111 (81.9)	2
Total number of hospital beds	588 (477–773)	628 (516–850)	18	601 (493–795)	8
Academic hospital	175,851 (33.2)	2,024 (42.8)	20	8,339 (37.7)	10
Annual volume of ambulance acceptance	4592 (2561)	4488 (2465)	− 4	4586 (2518)	0
Tertiary emergency hospital	310,967 (58.6)	2,892 (61.2)	5	12,895 (58.3)	− 1

*CNL* care-needs level, *ICU* intensive care unit, *IMCU* intermediate care unit, *SMD* standardized mean difference, *SOFA* Sequential Organ Failure Assessment, *SL* support level

Third, we visualized hospital-level ICU readmission rates using histograms to examine hospital-level variability in ICU readmission rates. Thereafter, we quantified between-hospital variation using the intraclass correlation coefficient (ICC), median odds ratio (MOR), and proportional change in variance (PCV) [22, 23]. Three hierarchical models were evaluated sequentially: model 1 included only random intercepts for hospital and quantified baseline variation between hospitals; patient-level variables were added to model 2 to assess how much of the variation between hospitals could be explained by patient characteristics; and model 3 further incorporated hospital-level variables to evaluate whether institutional characteristics accounted for the remaining variability. If substantial variation persisted after adjustment in model 3, it can be inferred that residual unmeasured factors influenced ICU readmission. An ICC of 0% indicates no cluster effect, whereas higher values, especially those above 10%, suggest substantial between-hospital variation; values approaching 100% imply that hospital-level factors predominantly determine ICU readmission [22]. The MOR represents the magnitude of the cluster effect on a familiar odds ratio scale (MOR = 1 indicates the lack of an effect, whereas larger or smaller values indicate greater variation) [22]. The PCV, the proportion of variance explained by adding patient- and hospital-level variables, was calculated as the difference between models 1 and 2, and between models 2 and 3.

Continuous variables were summarized as means with standard deviation (SD) or medians with interquartile ranges (IQRs), as appropriate. Categorical variables were expressed as frequencies and percentages. Statistical significance was set at a two-sided p-value < 0.05. All analyses were performed using Stata/SE software (version 19.0; StataCorp, College Station, TX, USA).

## Results

**Fig. 1:**
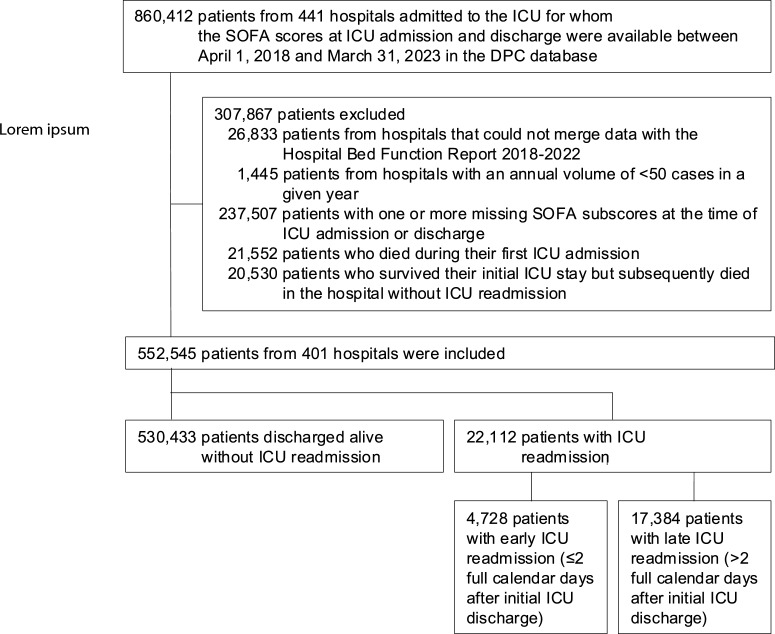
Flow chart of patient selection. *DPC* Diagnosis Procedure Combination *ICU* intensive care unit, *SOFA* Sequential Organ Failure Assessment

**Fig. 2:**
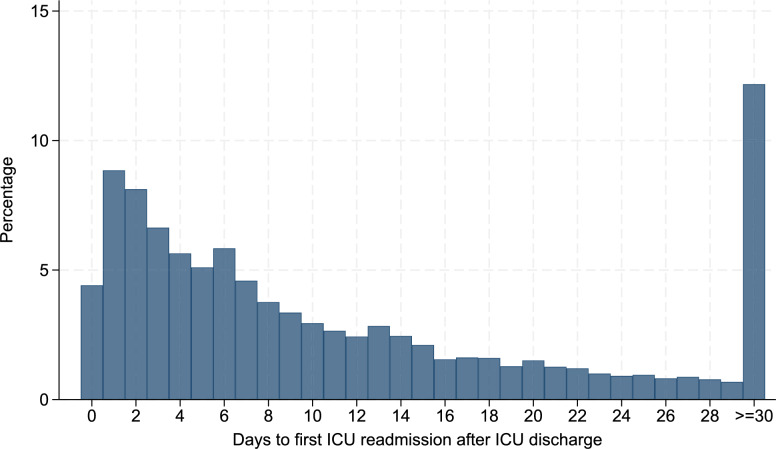
Histogram showing the distribution of days to first ICU readmission after the initial ICU discharge during the same hospitalization. The x-axis represents the number of days since ICU discharge (grouped into daily intervals up to ≥ 30 days), while the y-axis shows the percentage of all readmissions that occurred on that day. *ICU* intensive care unit

A total of 552,545 patients from 401 hospitals hospitalized between April 1, 2018, and March 31, 2023 were eligible for the analysis (Fig. 1). In fiscal year 2022, 6,132 ICU beds in Japan were reimbursed under codes A3011–A3014; of these, our study encompassed 3872 ICU beds (63%), reflecting broad national coverage. Overall, 22,112 (4.0%) patients (4.0%) were readmitted to the ICU during the same hospitalization and 4,728 patients (0.9% overall; 21.4% of all readmissions) experienced early ICU readmission (≤ 2 days). The distribution of days from the initial ICU discharge to the first ICU readmission did not exhibit a pronounced early peak; only a modest proportion of readmissions occurred within the first 2 calendar days, with the frequencies gradually declining thereafter (Fig. 2). Notably, a substantial share of readmissions occurred long after discharge: 32.8% of all ICU readmissions occurred ≥ 14 days after ICU discharge, and 12.2% occurred ≥ 30 days after discharge.

Compared with patients discharged alive without ICU readmission, those with early ICU readmission were more likely to have a higher Charlson Comorbidity Index, be admitted for emergency surgery, and have higher SOFA scores at both ICU admission and discharge (Table 1). Patients readmitted to the ICU were less likely to undergo elective surgery or have cancer as the primary Patients with early ICU readmission were more frequently treated in larger hospitals with more ICU and total beds and were more often admitted to academic hospitals.

The in-hospital mortality rate for early ICU readmissions was 18.8% (Table 2). These patients also had markedly longer total hospital stays and higher hospitalization costs. At ICU discharge, 13.5% of patients received any life-sustaining therapy, and 3.8% remained on therapy for ≥ 2 consecutive days including the day of discharge.

**Table 2:** Patient outcomes and life-sustaining therapies by ICU readmission status

Variables	Overall N = 552,545	Patients discharged alive without ICU readmission n = 530,433	Patients with early ICU readmission n = 4728	SMD (non-readmission vs. early readmission)	Patients with all ICU readmission n = 22,112	SMD (non-readmission vs. all readmission)
Outcomes						
ICU readmission	22,112 (4.0)	0 (0.0)	4,728 (100.0)	–	22,112 (100.0)	–
Early ICU readmission	4,728 (0.9)	0 (0.0)	4,728 (100.0)	–	4,728 (21.4)	–
Late ICU readmission	17,384 (3.1)	0 (0.0)	0 (0.0)	–	17,384 (78.6)	–
In-hospital mortality	4,735 (0.9)	0 (0.0)	889 (18.8)	–	4,735 (21.4)	–
Death during second ICU stay	1,952 (0.4)	0 (0.0)	372 (7.9)	–	1,952 (8.8)	–
Length of second ICU stay	3.0 (2.0–7.0)	–	4.0 (2.0–8.0)	–	3.0 (2.0–7.0)	–
Total length of hospital stay	19.0 (12.0–32.0)	18.0 (12.0–30.0)	36.0 (22.0–60.0)	58	51.0 (31.0–85.0)	80
Total hospitalization costs	2.4 (1.6–4.2)	2.3 (1.6–4.0)	4.9 (3.2–7.4)	77	6.1 (4.0–9.0)	95
Life-sustaining therapy during ICU stay						
Any life-sustaining therapy	278,068 (50.3)	259,538 (48.9)	4,051 (85.7)	85	18,530 (83.8)	79
Invasive mechanical ventilation	149,312 (27.0)	135,272 (25.5)	3,190 (67.5)	93	14,040 (63.5)	83
Noradrenaline	204,468 (37.0)	189,641 (35.8)	3,167 (67.0)	66	14,827 (67.1)	66
Adrenaline	34,461 (6.2)	31,723 (6.0)	568 (12.0)	21	2,738 (12.4)	22
Cardiopulmonary resuscitation	5,495 (1.0)	4,252 (0.8)	319 (6.8)	32	1,243 (5.6)	28
Mechanical circulatory support	17,147 (3.1)	15,235 (2.9)	437 (9.2)	27	1,912 (8.6)	25
Renal replacement therapy	33,447 (6.1)	28,371 (5.3)	1,037 (21.9)	50	5,076 (23.0)	52
Life-sustaining therapy on the day of ICU discharge						
Any life-sustaining therapy	74,354 (13.5)	68,930 (13.0)	1,517 (32.1)	47	5,424 (24.5)	30
Invasive mechanical ventilation	39,101 (7.1)	36,143 (6.8)	932 (19.7)	39	2,958 (13.4)	22
Noradrenaline	21,705 (3.9)	20,192 (3.8)	501 (10.6)	27	1,513 (6.8)	14
Adrenaline	1,800 (0.3)	1,700 (0.3)	51 (1.1)	9	100 (0.5)	2
Cardiopulmonary resuscitation	177 (0.0)	141 (0.0)	31 (0.7)	11	36 (0.2)	4
Mechanical circulatory support	1,627 (0.3)	1,491 (0.3)	60 (1.3)	11	136 (0.6)	5
Renal replacement therapy	18,672 (3.4)	16,962 (3.2)	380 (8.0)	21	1,710 (7.7)	20
Life-sustaining therapy for ≥ 2 consecutive days from the day of ICU discharge						
Any life-sustaining therapy	22,137 (3.8)	18,644 (3.5)	927 (19.6)	52	2,493 (11.3)	30
Invasive mechanical ventilation	13,127 (2.4)	11,535 (2.2)	607 (12.8)	41	1,592 (7.2)	24
Noradrenaline	4,740 (0.9)	4,062 (0.8)	306 (6.5)	31	678 (3.1)	17
Adrenaline	51 (0.0)	36 (0.0)	12 (0.3)	7	15 (0.1)	3
Cardiopulmonary resuscitation	0 (0.0)	0 (0.0)	0 (0.0)	–	0 (0.0)	–
Mechanical circulatory support	322 (0.1)	252 (0.0)	36 (0.8)	11	70 (0.3)	6
Renal replacement therapy	4,669 (0.8)	4,160 (0.8)	171 (3.6)	19	509 (2.3)	12

*ICU* intensive care unit, *SMD* standardized mean difference

Life-sustaining therapies included invasive mechanical ventilation, vasopressors (noradrenaline or adrenaline), cardiopulmonary resuscitation, mechanical circularity support, and renal replacement therapies

Late ICU readmissions were more likely to follow emergency surgery, have lower SOFA scores at ICU discharge, and were less likely to have circulatory or respiratory dysfunction at ICU discharge compared with early ICU readmissions (Supplementary Table 2). The in-hospital mortality rate for late readmissions was 22.1% versus 18.8% for early readmissions (SMD = 8%).

Among patients with early ICU readmission, the median total SOFA score increased from 4.0 (IQR 2.0–6.0) at first ICU discharge to 6.0 at ICU readmission (IQR 3.0–9.0; Supplementary Table 3). Respiratory and circulatory subscores showed the largest increases between discharge and readmission.

We conducted a supplementary analysis comparing patients who died in the hospital without ICU readmission (n = 20,530) with those who were readmitted to the ICU (n = 22,112; Supplementary Table 4). Patients who died without readmission were older, had greater long-term care needs, and showed greater disease severity at ICU discharge [median SOFA score 6.0 (IQR 4.0–9.0) vs. 3.0 (IQR 2.0–5.0)]. Nearly half of the patients (48.9%) were still receiving life-sustaining therapy at discharge, and 36.7% continued such therapy for ≥ 2 consecutive days.

Furthermore, we compared post-ICU transfers to IMCUs versus post-ICU transfers to general wards. Patients transferred to IMCUs had greater illness severity and received ongoing organ support more frequently at ICU discharge (Supplementary Table 5).

Multilevel logistic regression (Table 3) showed that male sex, higher Charlson Comorbidity, emergency surgery, non-surgery, certain diagnoses (e.g., aortic dissection/aneurysm, acute abdominal disease, sepsis), and fiscal year 2020–2022 were associated with higher odds of early ICU readmission. The SOFA score at ICU admission was not significantly associated with the risk of readmission. In contrast, higher SOFA scores at ICU discharge and residual respiratory or circulatory dysfunction were associated with higher rates of early ICU readmission. Shorter ICU stay (2–6 days), step-down transfer to an IMCU, and treatment in units reimbursed as A3013 or A3014 were associated with lower odds of early ICU readmission. The direction and magnitude of the hazard ratios derived from the sensitivity analysis using cause-specific Cox regression were comparable to the odds ratios derived from the main multilevel logistic regression (Supplementary Table 6).

**Table 3:** Multilevel logistic regression of the risk factors for early (≤ 2 days) and all ICU readmissions

Characteristics	Non-readmission vs. early readmissions, odds ratios (95% CIs)	P value	Non-readmission vs. all readmissions, odds ratios (95% CIs)	P value
Patient characteristics				
Age, years	1.00 (1.00–1.00)	0.341	1.00 (1.00–1.00)	0.719
Male	1.09 (1.02–1.16)	0.009	1.18 (1.15–1.22)	0.000
Charlson Comorbidity Index	1.05 (1.03–1.07)	0.000	1.07 (1.06–1.08)	0.000
Long-term care needs				
No care needs	Ref	–	Ref	–
SL1-2 and CNL1	1.12 (0.99–1.28)	0.073	1.29 (1.22–1.37)	0.000
CNL 2–5	1.03 (0.90–1.18)	0.666	1.14 (1.07–1.21)	0.000
Admission classification				
Elective surgery	Ref	–	Ref	–
Emergency surgery	2.06 (1.90–2.24)	0.000	2.77 (2.67–2.88)	0.000
Non-surgery	1.26 (1.16–1.37)	0.000	1.05 (1.01–1.10)	0.017
Primary diagnosis category				
Cancer	1.15 (1.06–1.26)	0.002	1.24 (1.19–1.29)	0.000
Acute coronary syndrome	1.05 (0.95–1.16)	0.336	1.01 (0.97–1.07)	0.576
Aortic dissection or aneurysm	1.32 (1.20–1.45)	0.000	1.44 (1.38–1.51)	0.000
Stroke	1.03 (0.90–1.19)	0.626	1.05 (0.98–1.11)	0.162
Acute abdominal diseases	1.30 (1.17–1.45)	0.000	1.13 (1.08–1.19)	0.000
Acute heart failure	1.21 (1.08–1.37)	0.002	1.51 (1.43–1.60)	0.000
Sepsis	1.28 (1.13–1.45)	0.000	1.44 (1.36–1.53)	0.000
Trauma	1.25 (1.06–1.47)	0.007	1.16 (1.07–1.25)	0.000
Total SOFA score at ICU admission	1.00 (0.99–1.02)	0.486	1.00 (1.00–1.01)	0.745
Total SOFA score at ICU discharge	1.17 (1.14–1.20)	0.000	1.10 (1.09–1.12)	0.000
SOFA subscore ≥ 2 at ICU discharge				
Respiratory	1.23 (1.14–1.34)	0.000	1.03 (0.99–1.07)	0.128
Platelet	1.00 (0.91–1.09)	0.923	1.00 (0.95–1.05)	0.969
Liver	0.96 (0.86–1.08)	0.509	1.12 (1.06–1.18)	0.000
Circulatory	1.25 (1.12–1.39)	0.000	1.10 (1.04–1.16)	0.001
Neurological	0.96 (0.86–1.08)	0.483	1.00 (0.94–1.05)	0.935
Renal	1.06 (0.95–1.19)	0.263	1.27 (1.21–1.34)	0.000
Length of first ICU stay				
1 day	Ref	–	Ref	–
2 days	0.67 (0.57–0.78)	0.000	0.72 (0.67–0.77)	0.000
3–6 days	0.82 (0.70–0.96)	0.015	1.08 (1.01–1.16)	0.030
≥ 7 days	1.03 (0.87–1.21)	0.760	1.59 (1.47–1.71)	0.000
Step-down transfer from ICU to IMCU	0.74 (0.66–0.82)	0.000	1.00 (0.95–1.05)	0.967
Fiscal year				
2018–2019	Ref	–	Ref	–
2020–2022	1.18 (1.09–1.27)	0.000	0.99 (0.96–1.03)	0.732
Season				
Spring	Ref	–	Ref	–
Summer	1.12 (1.03–1.22)	0.008	1.04 (1.00–1.09)	0.033
Fall	1.11 (1.02–1.21)	0.014	1.05 (1.01–1.09)	0.013
Winter	0.99 (0.91–1.07)	0.744	0.94 (0.90–0.98)	0.003
Weekend discharge from ICU	0.94 (0.87–1.02)	0.144	0.91 (0.88–0.94)	0.000
Hospital characteristics				
Reimbursement under ICU code				
A3011	Ref	–	Ref	–
A3012	0.97 (0.80–1.17)	0.738	0.86 (0.77–0.96)	0.005
A3013	0.69 (0.59–0.80)	0.000	0.79 (0.72–0.86)	0.000
A3014	0.74 (0.58–0.94)	0.012	0.76 (0.66–0.88)	0.000
Number of ICU beds	1.03 (1.02–1.03)	0.000	1.01 (1.00–1.01)	0.001
Hospital with IMCU beds	0.94 (0.79–1.12)	0.496	0.88 (0.79–0.98)	0.017
Total number of hospital beds	1.00 (1.00–1.00)	0.102	1.00 (1.00–1.00)	0.251
Academic hospital	1.11 (0.87–1.42)	0.417	0.98 (0.82–1.17)	0.823
Annual volume of ambulance acceptance	1.00 (1.00–1.00)	0.888	1.00 (1.00–1.00)	0.129
Tertiary emergency hospital	0.85 (0.70–1.02)	0.078	1.08 (0.96–1.23)	0.202

*CNL* care-needs level, *CI* confidence interval, *ICU* intensive care unit, *IMCU* intermediate care unit, *IMV* invasive mechanical ventilation, *OR* odds ratio, *RRT* renal replacement therapy, *SOFA* Sequential Organ Failure Assessment, *SL* support level

Comparison of the predictors of early versus all ICU readmissions (Table 3) revealed several differences. At ICU discharge, hepatic and renal SOFA subscores newly emerged as risk factors for all readmissions, whereas circulatory dysfunction lost its significance. In addition, step-down transfer to an IMCU, which was protective against early readmission, lost its protective association when all readmissions were considered.

**Fig. 3:**
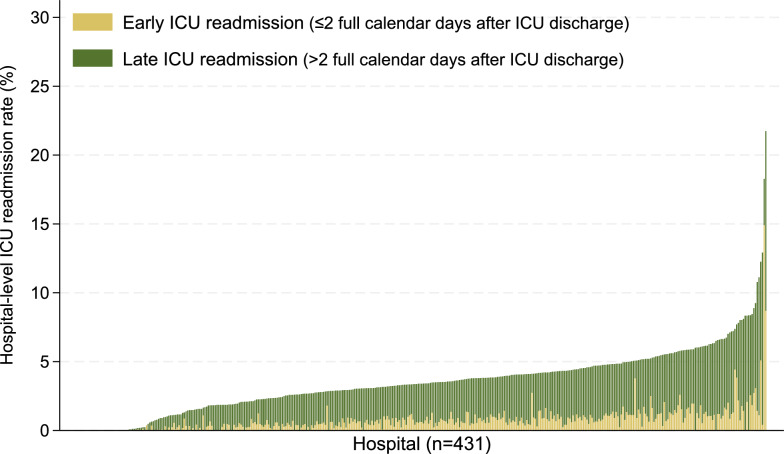
Hospital-level variation in ICU readmission rates among 431 hospitals. The bars represent readmission rates (%) by hospital, stratified by early readmissions (≤ 2 full calendar days after ICU discharge, yellow bars), and late readmissions (> 2 full calendar days, green bars). *ICU* intensive care unit

Hospital-level ICU readmission rates varied widely across facilities (Fig. 3), with median rates of 0.61% (IQR 0.25–1.00%, 99th percentile, 3.8%) for early readmissions and 3.4% (IQR 2.1–4.7%, 99th percentile, 11.1%) for all readmissions.

In the hierarchical models, early ICU readmission showed substantial between-hospital variation in model 1 (ICC = 14.6%, MOR = 2.04; Table 4). The addition of patient-level covariates to model 2 produced virtually no change in variability (ICC = 14.8%, MOR = 2.05, PCV = − 1.7%). Similarly, the variance remained unchanged after inclusion of hospital-level characteristics in model 3, (ICC = 13.9%, MOR = 2.05, PCV = 7.2%), indicating persistent unexplained hospital-level heterogeneity. The results for all ICU readmissions were similar.

**Table 4:** Between-hospital variation in ICU readmission: hierarchical model metrics (ICC, MOR, and PCV) across sequential models

Statistic	Model 1	Model 2	Model 3
Model parameter			
Fixed effect	None	Patient characteristics	Patient + hospital characteristics
Random effect	Random intercepts for hospital	Random intercepts for hospital	Random intercepts for hospital
vs. early ICU readmission			
ICC (%)	14.6 (12.1, 17.6)	14.8 (12.3, 17.8)	13.9 (11.5, 16.8)
MOR	2.04 (1.88, 2.20)	2.05 (1.90–2.22)	2.05 (1.91–2.23)
PCV (%)			
Models 1 and 2	Ref	− 1.7	–
Models 2 and 3	–	Ref	7.2
vs. all ICU readmission			
ICC (%)	14.7 (12.5, 17.2)	13.9 (11.8, 16.3)	13.3 (11.3, 15.8)
MOR	2.05 (1.92, 2.20)	2.00 (1.88, 2.14)	1.98 (1.85, 2.12)
PCV (%)			
Models 1 and 2	Ref	0.8	–
Models 2 and 3	–	Ref	4.2

*ICC* intraclass correlation coefficient, *ICU* intensive care unit, *MOR* median odds ratio, *PCV* proportional change in variance

## Discussion

In this nationwide study encompassing over half a million ICU admissions across 401 Japanese hospitals, ICU readmission occurred in 4.0% of patients, but early readmission within 2 full calendar days was uncommon (0.9% overall; 21.4% of all readmissions) and lacked a pronounced early peak, with a substantial proportion occurring long after discharge (≥ 14 days, 32.8%; ≥ 30 days, 12.2%). The SOFA scores at discharge, especially residual respiratory and circulatory dysfunction, were strong predictors of early readmission. The protective effects of IMCU transfer for early readmission disappeared when all readmissions were considered. Hospital-level variation remained substantial (ICC: 14–15%), even after adjustment for patient- and hospital-level factors.

The early ICU readmission rate in Japan (0.9%) is less than half of that reported by most U.S. and European studies (typically 2–3%) [8–14], suggesting potential differences in patient mix, discharge practices, and system-level factors. First, Japanese ICUs admit a relatively high proportion of elective surgical patients compared with their Western counterparts (52.9%, 31.2%, and 44.9% in the national ICU registries of Japan, England, Australia, and New Zealand, respectively) [24–26], which may mitigate physiological instability at discharge. Second, only a small proportion of patients received life-sustaining therapy on and the day after ICU discharge. When a stricter definition— continuation of therapy for ≥ 2 consecutive days from the day of ICU discharge—was applied, only 3.8% met this criterion, indicating that actual ongoing life-sustaining therapy at ICU discharge was uncommon. Third, IMCU beds are widely available in Japan [27]. In our analysis, step-down transfer to an IMCU was independently associated with a lower risk of early ICU readmission, and IMCUs managed more severely ill patients receiving ongoing organ support at discharge. These findings suggest that IMCUs function as an effective buffer between ICUs and general wards and may help reduce ICU readmissions. Fourth, compared with previous American and European studies [9, 10], the proportion of late ICU readmissions was greater in Japan, with 32.8% occurring ≥ 14 days and 12.2% ≥ 30 days after discharge. This may inflate the overall readmission rate, suggesting that Japan’s low early readmission rate reflects fewer total readmissions. Fifth, it is possible that the low early readmission rate partially reflects patients whose condition deteriorated unexpectedly but were not readmitted to the ICU. In our study, 3.6% (n = 20,530/573,075) of patients who survived the initial ICU stay died later in the hospital without ICU readmission. This proportion is comparable to that reported in previous studies from the United States (5.0%) and Australia (4.4%) [7, 10]. Our supplementary analysis revealed that nearly half (48.9%) of the patients remained on life-sustaining therapy at ICU discharge, suggesting that many of these deaths occurred among patients with persistently severe illness rather than those with unrecognized deterioration. Although it is unclear whether these patients should have been readmitted, in-hospital deaths without ICU readmission were not more frequent in Japan than in other countries. Further international comparative studies are needed to clarify the mechanisms underlying Japan’s lower rate of early ICU readmission.

The risk factors for ICU readmission in our study showed important differences from those reported in previous studies. In contrast to a previous Japanese ICU study with a smaller sample population, which found that severity at ICU admission (APACHE III score) was associated with the risk of readmission [16], our analysis found no significant association between the SOFA scores at ICU admission and ICU readmission. Instead, the SOFA scores at discharge, particularly residual respiratory and circulatory dysfunction—emerged as key predictors. This underscores the importance of assessing physiological stability at the time of ICU discharge rather than relying solely on disease severity at admission to guide discharge decisions. The prominence of residual respiratory and circulatory impairments is consistent with previous international findings [28, 29]; however, our results underscore the fact that some patients are still discharged from Japanese ICUs, suggesting a need to reevaluate discharge practices in Japan. Early and overall ICU readmissions likely represent different phases of clinical deterioration: early events reflect premature discharge, overall readmissions capture later, unrelated deterioration. Notably, step-down transfer to an IMCU, which was protective against early readmission, was no longer protective when all readmissions were considered. These findings suggest that early and overall readmissions have partly different determinants and should be interpreted as related but distinct quality indicators.

In our hierarchical models, hospital-level variation in ICU readmission remained substantial even after risk adjustment: for early readmission, the ICC was 13.9–14.8% with an MOR of approximately 2.0, and the ICC for all readmissions was 13.3–14.7% with similar MOR values. These figures indicate that two otherwise similar patients discharged from two randomly selected hospitals could face nearly a twofold difference in the risk of readmission solely due to institutional factors. Such persistent variability, even after adjusting for a comprehensive set of patient- and hospital-level characteristics, suggests the presence of unmeasured hospital-specific practices or resources—possibly differences in facility-specific discharge and readmission criteria—that influence the risk of readmission. This magnitude of unexplained variation raises concerns about using ICU readmission rates as a stand-alone quality indicator: the high ICC values imply that differences in readmission frequency may reflect structural or operational heterogeneity between hospitals rather than true differences in the quality of care. Therefore, while ICU readmission can provide useful contextual information, our findings support cautious interpretation and combining readmission rates with other performance measures when benchmarking ICU quality [1–4].

The strength of this study lies in its recruitment of a vast nationwide inpatient database, which provides a nationally representative cohort covering approximately 60% of all ICU beds reimbursed under codes A3011–A3014. Furthermore, the use of discharge SOFA scores—rather than the admission scores—facilitated evaluation of physiological stability at the critical point of ICU discharge, providing important insights into discharge practices. However, this study had some limitations. First, our administrative data limited the granularity of the information available for analysis; for example, we lacked data on the exact timestamp of ICU discharge, socioeconomic factors, and detailed post-ICU monitoring protocols. Second, the definition, organization, staffing, equipment, interventions, patient case-mix, and utilization of ICUs vary among countries. Since this study presents findings from a single country, the results may not be generalizable to other countries. Third, our analysis was conducted at the hospital level rather than at the ICU level. Therefore, in cases where a single hospital had multiple independent ICUs, we were unable to perform analyses that accounted for the differences among these individual units. Fourth, this study focused on associations rather than causality, and results such as the association between IMCU availability and early readmission should be construed accordingly. Fifth, our findings should be interpreted with caution because emergency and critical care units (medical reimbursement codes: A3002 and A3004) were excluded. These units, which mainly manage emergency medical and non-surgical cases, are expected to have different patterns and higher risks of ICU readmission.

## Conclusion

This nationwide study demonstrated that 4.0% of patients underwent ICU readmission, with early readmission being rare (0.9%) and several readmissions occurring late after ICU discharge. The SOFA scores at discharge, especially residual respiratory and circulatory dysfunction, predicted ICU readmission, whereas disease severity at admission and ICU length of stay did not. Persistent between-hospital variability indicates strong institutional influence and limits the utility of ICU readmission rates as a standalone indicator of the quality of care.

## Supplementary Information

Supplementary material 1.

## Data Availability

The dataset analyzed in the current study is not publicly available due to contracts with the hospitals that provide data to the database.

## Abbreviations

APACHE: Acute physiology and chronic health evaluation
CI: Confidence interval
DPC: Diagnosis procedure combination
ICU: Intensive care unit
ICC: Intraclass correlation coefficient
IMCU: Intermediate care unit
IQR: Interquartile range
JIPAD: Japanese intensive care patient database
MOR: Median odds ratio
OR: Odds ratio
PCV: Proportional change in variance
SD: Standard deviation
SMD: Standardized mean difference
SOFA: Sequential organ failure assessment
