# Supplementary material for: Predictors of and hospital-level variation in intensive care unit readmissions in Japan: a nationwide inpatient database study
Source: J Intensive Care. 2025 Nov 29;14:2. doi: 10.1186/s40560-025-00838-3 (PMC12771954; doi:10.1186/s40560-025-00838-3)
Supplement: Supplementary file 1 — Supplementary material 1. [file 40560_2025_838_MOESM1_ESM.docx]

**Supplementary Materials**

**Predictors of and Hospital-Level Variation in Intensive Care Unit Readmissions in Japan: A Nationwide Inpatient Database Study**

Hiroyuki Ohbe^1,2^**^*^**, Yusuke Sasabuchi^3^, Yuya Kimura^4^, Hiroki Matsui^2^, Kiyohide Fushimi^5^, Hideo Yasunaga^2^, Daisuke Kudo^1,6^

^1^Department of Emergency and Critical Care Medicine, Tohoku University Hospital, 1-1 Seiryo-machi, Aoba-ku, Sendai 980-8574, Japan

^2^Department of Real-world Evidence, The Graduate School of Medicine, The University of Tokyo, 7-3-1 Hongo, Bunkyo-ku, Tokyo 113-0033, Japan

^3^Department of Clinical Epidemiology and Health Economics, School of Public Health, The University of Tokyo, 7-3-1 Hongo, Bunkyo-ku, Tokyo 113-0033, Japan

^4^Department of Health Services Research, Graduate School of Medicine, The University of

Tokyo, 7-3-1 Hongo, Bunkyo-ku, Tokyo 113-0033, Japan

^5^Department of Health Policy and Informatics, Institute of Science, Tokyo Graduate School, 2-12-1 Ookayama, Meguro-ku, Tokyo 152-8550, Japan

^6^Division of Emergency and Critical Care Medicine, Tohoku University Graduate School of Medicine, 2-1 Seiryo-machi, Aoba-ku, Sendai, Miyagi, 980-8575, Japan

**List of Supplementary Tables**

**Supplementary Table 1.** Japanese medical procedure codes used to define the ICU in 2022

ICU: intensive care unit

**Supplementary Table 2.** Patient characteristics, hospital characteristics, and outcomes of early and late ICU readmissions

ICU: intensive care unit

**Supplementary Table 3.** Changes in total and organ-specific SOFA scores between the first ICU discharge and second ICU admission

**Supplementary Table 4.** Patient characteristics, hospital characteristics, and outcomes in patients with ICU readmission versus those who died in-hospital without ICU readmission

**Supplementary Table 5.** Patient characteristics, hospital characteristics, and outcomes in patients transferred to IMCUs versus general wards after ICU discharge

**Supplementary Table 6.** Time-to-event analysis using a cause-specific Cox proportional hazards model for the risk factors for early (≤2 days) and all ICU readmissions

**Supplementary Table 1.** Japanese medical procedure codes used to define the ICU in 2022

|  |  |  | Cost |  |  |  |  |  |  |
| --- | --- | --- | --- | --- | --- | --- | --- | --- | --- |
|  |  |  | per day, |  | Non- | Night-shift | Nurse to | ICU | Clinical |
| Type | Code | Description | JPY* | Intensivist | intensivist | physician | patient ratio | Nurse** | Engineer |
| ICU | A3011 | ICU management fee 1 | 142,110 | ≥2 in ICU 24/7 | – | Dedicated to ICU | 1:2 | Required | 24/7 in hospital |
| ICU | A3012 | ICU management fee 2 | 142,110 | ≥2 in ICU 24/7 | – | Dedicated to ICU | 1:2 | Required | 24/7 in hospital |
| ICU | A3013 | ICU management fee 3 | 96,970 | – | ≥1 in ICU 24/7 | Dedicated to ICU | 1:2 | – | – |
| ICU | A3014 | ICU management fee 4 | 96,970 | – | ≥1 in ICU 24/7 | Dedicated to ICU | 1:2 | – | – |

*Cost per day for the first 7 days after admission in 2022

**A full-time ICU nurse is defined as one who works at least 20 h per week in the ICU.

ICU: intensive care unit, IMCU: intermediate care unit

**Supplementary Table 2.** Patient characteristics, hospital characteristics, and outcomes of early and late ICU readmissions

|  | Patients with | Patients with |  |
| --- | --- | --- | --- |
|  | early ICU | late ICU |  |
|  | readmission | readmission | SMD |
| Characteristics | N=4,728 | N=17,384 | % |
| **Patient characteristics** |  |  |  |
| Age, years | 69.6 (14.2) | 69.5 (14.5) | -1 |
| Male | 3,076 (65.1) | 11,507 (66.2) | 2 |
| Charlson Comorbidity Index | 1.5 (1.6) | 1.5 (1.7) | 2 |
| Long-term care needs, n (%) |  |  |  |
| No care needs | 4,169 (88.2) | 15,126 (87.0) | -4 |
| SL1-2 and CNL1 | 293 (6.2) | 1,194 (6.9) | 3 |
| CNL 2-5 | 266 (5.6) | 1,064 (6.1) | 2 |
| Admission classification |  |  |  |
| Elective surgery | 1,835 (38.8) | 5,861 (33.7) | -11 |
| Emergency surgery | 1,411 (29.8) | 7,198 (41.4) | 24 |
| Non-surgery | 1,482 (31.3) | 4,325 (24.9) | -14 |
| Primary diagnosis category |  |  |  |
| Cancer | 996 (21.1) | 3,747 (21.6) | 1 |
| Acute coronary syndrome | 580 (12.3) | 1,880 (10.8) | -5 |
| Aortic dissection or aneurysm | 755 (16.0) | 3,127 (18.0) | 5 |
| Stroke | 276 (5.8) | 1,242 (7.1) | 5 |
| Acute abdominal diseases | 530 (11.2) | 2,035 (11.7) | 2 |
| Acute heart failure | 366 (7.7) | 1,532 (8.8) | 4 |
| Sepsis | 349 (7.4) | 1,392 (8.0) | 2 |
| Trauma | 178 (3.8) | 728 (4.2) | 2 |
| Total SOFA score at ICU admission | 5.0 (2.0-8.0) | 5.0 (2.0-8.0) | -7 |
| Total SOFA score at ICU discharge | 4.0 (2.0-6.0) | 3.0 (1.0-5.0) | -18 |
| SOFA subscore ≥2 at ICU discharge |  |  |  |
| Respiratory | 1,735 (36.7) | 5,152 (29.6) | -15 |
| Platelet | 1,035 (21.9) | 3,117 (17.9) | -10 |
| Liver | 490 (10.4) | 1,687 (9.7) | -2 |
| Circulatory | 865 (18.3) | 1,939 (11.2) | -20 |
| Neurological | 681 (14.4) | 2,348 (13.5) | -3 |
| Renal | 908 (19.2) | 3,324 (19.1) | 0 |
| Length of first ICU stay |  |  |  |
| 1 day | 265 (5.6) | 1,149 (6.6) | 4 |
| 2 days | 1,729 (36.6) | 5,240 (30.1) | -14 |
| 3–6 days | 1,771 (37.5) | 6,425 (37.0) | -1 |
| ≥7 days | 963 (20.4) | 4,570 (26.3) | 14 |
| Step-down transfer from ICU to IMCU | 528 (11.2) | 2,270 (13.1) | 6 |
| Fiscal year |  |  |  |
| 2018–2019 | 1,316 (27.8) | 5,040 (29.0) | 3 |
| 2020–2022 | 3,412 (72.2) | 12,344 (71.0) | 3 |
| Season |  |  |  |
| Spring | 1,066 (22.5) | 4,119 (23.7) | -3 |
| Summer | 1,213 (25.7) | 4,311 (24.8) | 2 |
| Fall | 1,288 (27.2) | 4,681 (26.9) | 1 |
| Winter | 1,161 (24.6) | 4,273 (24.6) | 0 |
| Weekend discharge from ICU | 775 (16.4) | 2,728 (15.7) | -2 |
| **Hospital characteristics** |  |  |  |
| Reimbursement under ICU code |  |  |  |
| A3011 | 2,398 (50.7) | 8,054 (46.3) | -9 |
| A3012 | 1,114 (23.6) | 4,262 (24.5) | 2 |
| A3013 | 920 (19.5) | 3,753 (21.6) | 5 |
| A3014 | 296 (6.3) | 1,315 (7.6) | 5 |
| Number of ICU beds | 16 (10-23) | 16 (10-22) | -11 |
| Hospital with IMCU beds | 3,868 (81.8) | 14,243 (81.9) | 0 |
| Total number of hospital beds | 628 (516-850) | 598 (489-792) | -12 |
| Academic hospital | 2,024 (42.8) | 6,315 (36.3) | -13 |
| Annual volume of ambulance acceptance | 4488 (2465) | 4612 (2531) | 5 |
| Tertiary emergency hospital | 2,892 (61.2) | 10,003 (57.5) | -7 |
| **Outcomes** |  |  |  |
| ICU readmission | 4,728 (100.0) | 17,384 (100.0) | – |
| In-hospital mortality | 889 (18.8) | 3,846 (22.1) | 8 |
| Death during second ICU stay, days | 372 (7.9) | 1,580 (9.1) | 4 |
| Length of second ICU stay, days | 4.0 (2.0-8.0) | 3.0 (2.0-7.0) | -7 |
| Total length of hospital stay, days | 36.0 (22.0-60.0) | 56.0 (35.0-91.0) | 38 |
| Total hospitalization costs, million yen | 4.9 (3.2-7.4) | 6.4 (4.2-9.3) | 29 |
| **Life-sustaining therapy during ICU stay** |  |  |  |
| Any life-sustaining therapy | 4,051 (85.7) | 14,479 (83.3) | 7 |
| Invasive mechanical ventilation | 3,190 (67.5) | 10,850 (62.4) | 11 |
| Noradrenaline | 3,167 (67.0) | 11,660 (67.1) | 0 |
| Adrenaline | 568 (12.0) | 2,170 (12.5) | -1 |
| Cardiopulmonary resuscitation | 319 (6.8) | 924 (5.3) | 6 |
| Mechanical circulatory support | 437 (9.2) | 1,475 (8.5) | 3 |
| Renal replacement therapy | 1,037 (21.9) | 4,039 (23.2) | -3 |
| **Life-sustaining therapy on the day of ICU discharge** |  |  |  |
| Any life-sustaining therapy | 1,517 (32.1) | 3,907 (22.5) | 22 |
| Invasive mechanical ventilation | 932 (19.7) | 2,026 (11.7) | 22 |
| Noradrenaline | 501 (10.6) | 1,012 (5.8) | 18 |
| Adrenaline | 51 (1.1) | 49 (0.3) | 10 |
| Cardiopulmonary resuscitation | 31 (0.7) | 5 (0.0) | 11 |
| Mechanical circulatory support | 60 (1.3) | 76 (0.4) | 9 |
| Renal replacement therapy | 380 (8.0) | 1,330 (7.7) | 1 |
| **Life-sustaining therapy for ≥2 consecutive** |  |  |  |
| **days from the day of ICU discharge** |  |  |  |
| Any life-sustaining therapy | 927 (19.6) | 1,566 (9.0) | 31 |
| Invasive mechanical ventilation | 607 (12.8) | 985 (5.7) | 25 |
| Noradrenaline | 306 (6.5) | 372 (2.1) | 22 |
| Adrenaline | 12 (0.3) | 3 (0.0) | 6 |
| Cardiopulmonary resuscitation | 0 (0.0) | 0 (0.0) | – |
| Mechanical circulatory support | 36 (0.8) | 34 (0.2) | 8 |
| Renal replacement therapy | 171 (3.6) | 338 (1.9) | 10 |

CNL: care-needs level, ICU: intensive care unit, IMCU: intermediate care unit, IQR: interquartile range, SD: standard deviation, SMD: standardized mean difference, SOFA: Sequential Organ Failure Assessment, SL: support level

**Supplementary Table 3.** Changes in total and organ-specific SOFA scores between the first ICU discharge and ICU readmission

|  | Early ICU readmission | |  | All ICU readmission | |
| --- | --- | --- | --- | --- | --- |
|  | At first ICU | At ICU |  | At first ICU | At ICU |
|  | discharge | readmission |  | discharge | readmission |
| Characteristics | n=4,728 | n=4,728 |  | n=22,112 | n=22,112 |
| Total SOFA score | 4.0 (2.0-6.0) | 6.0 (3.0-9.0) |  | 3.0 (2.0-5.0) | 5.0 (3.0-9.0) |
| SOFA subscore ≥2 |  |  |  |  |  |
| Respiratory | 1,735 (36.7) | 2,501 (55.5) |  | 6,887 (31.1) | 10,156 (48.3) |
| Platelet | 1,035 (21.9) | 1,103 (24.2) |  | 4,152 (18.8) | 3,928 (18.6) |
| Liver | 490 (10.4) | 568 (12.6) |  | 2,177 (9.8) | 2,257 (10.8) |
| Circulatory | 865 (18.3) | 1,607 (34.8) |  | 2,804 (12.7) | 7,164 (33.2) |
| Neurological | 681 (14.4) | 1,658 (36.0) |  | 3,029 (13.7) | 8,058 (37.5) |
| Renal | 908 (19.2) | 1,043 (22.9) |  | 4,232 (19.1) | 4,954 (23.4) |

ICU: intensive care unit, SOFA: Sequential Organ Failure Assessment

**Supplementary Table 4.** Patient characteristics, hospital characteristics, and outcomes in patients with ICU readmission versus those who died in-hospital without ICU readmission

|  | Patients with | Patients who died |  |  |
| --- | --- | --- | --- | --- |
|  | all ICU | in-hospital without |  |  |
|  | readmission | ICU readmission |  |  |
| Characteristics | n=22,112 | n=20,530 | SMD |  |
| **Patient characteristics** |  |  |  |  |
| Age, years | 69.5 (14.5) | 74.4 (13.4) | -35 |  |
| Male | 14,583 (66.0) | 12,986 (63.3) | 6 |  |
| Charlson Comorbidity Index | 1.5 (1.6) | 1.7 (1.9) | -11 |  |
| Long-term care needs, n (%) |  |  |  |  |
| No care needs | 19,295 (87.3) | 15,689 (76.4) | 28 |  |
| SL1-2 and CNL1 | 1,487 (6.7) | 1,949 (9.5) | -10 |  |
| CNL 2-5 | 1,330 (6.0) | 2,892 (14.1) | -27 |  |
| Admission classification |  |  |  |  |
| Elective surgery | 7,696 (34.8) | 1,447 (7.0) | 73 |  |
| Emergency surgery | 8,609 (38.9) | 4,948 (24.1) | 32 |  |
| Non-surgery | 5,807 (26.3) | 14,135 (68.9) | -94 |  |
| Primary diagnosis category |  |  |  |  |
| Cancer | 4,743 (21.4) | 3,963 (19.3) | 5 |  |
| Acute coronary syndrome | 2,460 (11.1) | 1,403 (6.8) | 15 |  |
| Aortic dissection or aneurysm | 3,882 (17.6) | 1,277 (6.2) | 36 |  |
| Stroke | 1,518 (6.9) | 2,239 (10.9) | -14 |  |
| Acute abdominal diseases | 2,565 (11.6) | 2,900 (14.1) | -8 |  |
| Acute heart failure | 1,898 (8.6) | 2,027 (9.9) | -4 |  |
| Sepsis | 1,741 (7.9) | 3,686 (18.0) | -30 |  |
| Trauma | 906 (4.1) | 995 (4.8) | -4 |  |
| Total SOFA score at ICU admission | 5.0 (2.0-8.0) | 7.0 (4.0-10.0) | -55 |  |
| Total SOFA score at ICU discharge | 3.0 (2.0-5.0) | 6.0 (4.0-9.0) | -79 |  |
| SOFA subscore ≥2 at ICU discharge |  |  |  |  |
| Respiratory | 6,887 (31.1) | 9,297 (45.3) | -29 |  |
| Platelet | 4,152 (18.8) | 6,396 (31.2) | -29 |  |
| Liver | 2,177 (9.8) | 3,396 (16.5) | -20 |  |
| Circulatory | 2,804 (12.7) | 4,806 (23.4) | -28 |  |
| Neurological | 3,029 (13.7) | 9,778 (47.6) | -79 |  |
| Renal | 4,232 (19.1) | 6,416 (31.3) | -28 |  |
| Length of first ICU stay |  |  |  |  |
| 1 day | 1,414 (6.4) | 909 (4.4) | 9 |  |
| 2 days | 6,969 (31.5) | 4,019 (19.6) | 28 |  |
| 3–6 days | 8,196 (37.1) | 6,995 (34.1) | 6 |  |
| ≥7 days | 5,533 (25.0) | 8,607 (41.9) | -36 |  |
| Step-down transfer from ICU to IMCU | 2,798 (12.7) | 3,375 (16.4) | -11 |  |
| Fiscal year |  |  |  |  |
| 2018–2019 | 6,356 (28.7) | 5,179 (25.2) | 8 |  |
| 2020–2022 | 15,756 (71.3) | 15,351 (74.8) | -8 |  |
| Season |  |  |  |  |
| Spring | 5,185 (23.4) | 4,807 (23.4) | 0 |  |
| Summer | 5,524 (25.0) | 4,662 (22.7) | 5 |  |
| Fall | 5,969 (27.0) | 5,227 (25.5) | 3 |  |
| Winter | 5,434 (24.6) | 5,834 (28.4) | -9 |  |
| Weekend discharge from ICU | 3,503 (15.8) | 3,080 (15.0) | 2 |  |
| **Hospital characteristics** |  |  |  |  |
| Reimbursed code of ICU |  |  |  |  |
| A3011 | 10,452 (47.3) | 7,759 (37.8) | 19 |  |
| A3012 | 5,376 (24.3) | 5,008 (24.4) | 0 |  |
| A3013 | 4,673 (21.1) | 5,619 (27.4) | -15 |  |
| A3014 | 1,611 (7.3) | 2,144 (10.4) | -11 |  |
| Number of ICU beds | 16.0 (10.0-22.0) | 12.0 (8.0-20.0) | 27 |  |
| Hospital with IMCU beds | 18,111 (81.9) | 16,933 (82.5) | -1 |  |
| Total number of hospital beds | 601.0 (493.0-795.0) | 573.0 (438.0-715.0) | 22 |  |
| Academic hospital | 8,339 (37.7) | 5,104 (24.9) | 28 |  |
| Annual volume of ambulance acceptance | 4585.5 (2517.5) | 4889.3 (2565.1) | -12 |  |
| Tertiary emergency hospital | 12,895 (58.3) | 11,625 (56.6) | 3 |  |
| **Outcomes** |  |  |  |  |
| ICU readmission | 22,112 (100.0) | 0 (0.0) | – |  |
| In-hospital mortality | 4,735 (21.4) | 20,530 (100.0) | – |  |
| Death during second ICU stay | 1,952 (8.8) | 0 (0.0) | – |  |
| Length of second ICU stay | 3.0 (2.0-7.0) |  |  |  |
| Total length of hospital stay | 51.0 (31.0-85.0) | 27.0 (14.0-51.0) | 33 |  |
| Total hospitalization costs | 6.1 (4.0-9.0) | 3.0 (1.7-5.3) | 54 |  |
| **Life-sustaining therapy during ICU stay** | |  |  |  |
| Any life-sustaining therapy | 18,530 (83.8) | 16,199 (78.9) | 13 |  |
| Invasive mechanical ventilation | 14,040 (63.5) | 12,600 (61.4) | 4 |  |
| Noradrenaline | 14,827 (67.1) | 12,035 (58.6) | 18 |  |
| Adrenaline | 2,738 (12.4) | 1,528 (7.4) | 17 |  |
| Cardiopulmonary resuscitation | 1,243 (5.6) | 1,781 (8.7) | -12 |  |
| Mechanical circulatory support | 1,912 (8.6) | 1,462 (7.1) | 6 |  |
| Renal replacement therapy | 5,076 (23.0) | 5,057 (24.6) | -4 |  |
| **Life-sustaining therapy on the day of ICU discharge** | |  |  |  |
| Any life-sustaining therapy | 5,424 (24.5) | 10,048 (48.9) | -52 |  |
| Invasive mechanical ventilation | 2,958 (13.4) | 7,317 (35.6) | -54 |  |
| Noradrenaline | 1,513 (6.8) | 3,967 (19.3) | -38 |  |
| Adrenaline | 100 (0.5) | 185 (0.9) | -6 |  |
| Cardiopulmonary resuscitation | 36 (0.2) | 102 (0.5) | -6 |  |
| Mechanical circulatory support | 136 (0.6) | 263 (1.3) | -7 |  |
| Renal replacement therapy | 1,710 (7.7) | 2,247 (10.9) | -11 |  |
| **Life-sustaining therapy for ≥2 consecutive**  **days from the day of ICU discharge** | | | | |
| Any life-sustaining therapy | 2,493 (11.3) | 7,529 (36.7) | -62 |  |
| Invasive mechanical ventilation | 1,592 (7.2) | 5,950 (29.0) | -59 |  |
| Noradrenaline | 678 (3.1) | 2,812 (13.7) | -39 |  |
| Adrenaline | 15 (0.1) | 79 (0.4) | -7 |  |
| Cardiopulmonary resuscitation | 0 (0.0) | 5 (0.0) | -2 |  |
| Mechanical circulatory support | 70 (0.3) | 197 (1.0) | -8 |  |
| Renal replacement therapy | 509 (2.3) | 812 (4.0) | -10 |  |

CNL: care-needs level, ICU: intensive care unit, IMCU: intermediate care unit, IQR: interquartile range, SD: standard deviation, SMD: standardized mean difference, SOFA: Sequential Organ Failure Assessment, SL: support level

**Supplementary Table 5.** Patient characteristics, hospital characteristics, and outcomes in patients transferred to IMCUs versus general wards after ICU discharge

|  | Patients | Patients |  |
| --- | --- | --- | --- |
|  | transferred to | transferred to |  |
|  | general wards | IMCUs |  |
| Characteristics | n=515,531 | n=57,544 | SMD |
| **Patient characteristics** |  |  |  |
| Age, years | 68.6 (14.9) | 69.4 (14.1) | 5 |
| Male | 314,208 (60.9) | 35,869 (62.3) | 3 |
| Charlson Comorbidity Index | 1.3 (1.6) | 1.3 (1.5) | 1 |
| Long-term care-needs, n (%) |  |  |  |
| No care needs | 464,222 (90.0) | 51,485 (89.5) | -2 |
| SL1-2 and CNL1 | 26,958 (5.2) | 2,978 (5.2) | 0 |
| CNL 2-5 | 24,351 (4.7) | 3,081 (5.4) | 3 |
| Admission classification |  |  |  |
| Elective surgery | 275,092 (53.4) | 23,567 (41.0) | -25 |
| Emergency surgery | 87,465 (17.0) | 13,760 (23.9) | 17 |
| Non-surgery | 152,974 (29.7) | 20,217 (35.1) | 12 |
| Primary diagnosis category |  |  |  |
| Cancer | 155,464 (30.2) | 8,689 (15.1) | -37 |
| Acute coronary syndrome | 55,006 (10.7) | 7,569 (13.2) | 8 |
| Aortic dissection or aneurysm | 58,765 (11.4) | 8,594 (14.9) | 10 |
| Stroke | 34,648 (6.7) | 6,815 (11.8) | 18 |
| Acute abdominal diseases | 39,314 (7.6) | 4,258 (7.4) | -1 |
| Acute heart failure | 27,193 (5.3) | 3,502 (6.1) | 4 |
| Sepsis | 19,753 (3.8) | 3,484 (6.1) | 10 |
| Trauma | 15,198 (2.9) | 1,977 (3.4) | 3 |
| Total SOFA score at ICU admission | 3.0 (1.0-6.0) | 5.0 (3.0-8.0) | 55 |
| Total SOFA score at ICU discharge | 2.0 (1.0-4.0) | 3.0 (2.0-6.0) | 48 |
| SOFA subscore ≥2 at ICU discharge |  |  |  |
| Respiratory | 108,907 (21.1) | 19,443 (33.8) | 29 |
| Platelet | 66,612 (12.9) | 12,075 (21.0) | 22 |
| Liver | 29,728 (5.8) | 5,098 (8.9) | 12 |
| Circulatory | 35,303 (6.8) | 9,595 (16.7) | 31 |
| Neurological | 41,521 (8.1) | 10,269 (17.8) | 29 |
| Renal | 52,190 (10.1) | 7,383 (12.8) | 8 |
| Length of first ICU stay |  |  |  |
| 1 day | 38,707 (7.5) | 2,730 (4.7) | -12 |
| 2 days | 261,213 (50.7) | 17,425 (30.3) | -42 |
| 3–6 days | 155,249 (30.1) | 25,200 (43.8) | 29 |
| ≥7 days | 60,362 (11.7) | 12,189 (21.2) | 26 |
| Step-down transfer from ICU to IMCU | 0 (0.0) | 57,544 (100.0) | – |
| Fiscal year |  |  |  |
| 2018–2019 | 134,521 (26.1) | 18,965 (33.0) | 15 |
| 2020–2022 | 381,010 (73.9) | 38,579 (67.0) | -15 |
| Season |  |  |  |
| Spring | 120,822 (23.4) | 13,492 (23.4) | 0 |
| Summer | 126,581 (24.6) | 14,012 (24.4) | 0 |
| Fall | 134,863 (26.2) | 14,970 (26.0) | 0 |
| Winter | 133,265 (25.9) | 15,070 (26.2) | 1 |
| Weekend discharge from ICU | 89,123 (17.3) | 10,678 (18.6) | 3 |
| **Hospital characteristics** |  |  |  |
| Reimbursed code of ICU |  |  |  |
| A3011 | 215,570 (41.8) | 28,943 (50.3) | 17 |
| A3012 | 117,089 (22.7) | 13,654 (23.7) | 2 |
| A3013 | 138,546 (26.9) | 10,859 (18.9) | -19 |
| A3014 | 44,326 (8.6) | 4,088 (7.1) | -6 |
| Number of ICU beds | 14.0 (8.0-22.0) | 15.0 (10.0-26.0) | 6 |
| Hospital with IMCU beds | 407,086 (79.0) | 57,332 (99.6) | 71 |
| Total number of hospital beds | 584.0 (477.0-763.0) | 638.0 (480.0-867.0) | 17 |
| Academic hospital | 168,866 (32.8) | 20,428 (35.5) | 6 |
| Annual volume of ambulance acceptance | 4565.5 (2541.1) | 4935.7 (2697.5) | 14 |
| Tertiary emergency hospital | 301,854 (58.6) | 33,633 (58.4) | 0 |
| **Outcomes** |  |  |  |
| ICU readmission | 19,314 (3.7) | 2,798 (4.9) | 6 |
| In-hospital mortality | 21,163 (4.1) | 4,102 (7.1) | 13 |
| Death during second ICU stay | 1,675 (0.3) | 277 (0.5) | 3 |
| Length of second ICU stay | 3.0 (2.0-7.0) | 4.0 (2.0-8.0) | 9 |
| Total length of hospital stay | 18.0 (12.0-31.0) | 25.0 (17.0-41.0) | 23 |
| Total hospitalization costs | 2.3 (1.6-4.0) | 3.5 (2.1-5.4) | 35 |
| **Life-sustaining therapy during ICU stay** | |  |  |
| Any life-sustaining therapy | 253,587 (49.2) | 40,680 (70.7) | 45 |
| Invasive mechanical ventilation | 132,266 (25.7) | 29,646 (51.5) | 55 |
| Noradrenaline | 186,299 (36.1) | 30,204 (52.5) | 33 |
| Adrenaline | 32,657 (6.3) | 3,332 (5.8) | -2 |
| Cardiopulmonary resuscitation | 5,885 (1.1) | 1,391 (2.4) | 10 |
| Mechanical circulatory support | 15,547 (3.0) | 3,062 (5.3) | 12 |
| Renal replacement therapy | 33,077 (6.4) | 5,427 (9.4) | 11 |
| **Life-sustaining therapy on the day of ICU discharge** | |  |  |
| Any life-sustaining therapy | 69,960 (13.6) | 14,442 (25.1) | 30 |
| Invasive mechanical ventilation | 36,886 (7.2) | 9,532 (16.6) | 29 |
| Noradrenaline | 21,554 (4.2) | 4,118 (7.2) | 13 |
| Adrenaline | 1,003 (0.4) | 182 (0.3) | 0 |
| Cardiopulmonary resuscitation | 239 (0.0) | 40 (0.1) | 1 |
| Mechanical circulatory support | 1,596 (0.3) | 294 (0.5) | 3 |
| Renal replacement therapy | 18,439 (3.6) | 2,480 (4.3) | 4 |
| **Life-sustaining therapy for ≥2 consecutive**  **days from the day of ICU discharge** | | | |
| Any life-sustaining therapy | 12,935 (4.3) | 6,731 (11.7) | 28 |
| Invasive mechanical ventilation | 14,233 (2.8) | 4,844 (8.4) | 25 |
| Noradrenaline | 5,878 (1.1) | 1,674 (2.9) | 13 |
| Adrenaline | 106 (0.0) | 24 (0.0) | 1 |
| Cardiopulmonary resuscitation | 4 (0.0) | 1 (0.0) | 0 |
| Mechanical circulatory support | 450 (0.1) | 69 (0.1) | 1 |
| Renal replacement therapy | 4,638 (0.9) | 843 (1.5) | 5 |

CNL: care-needs level, ICU: intensive care unit, IMCU: intermediate care unit, IQR: interquartile range, SD: standard deviation, SMD: standardized mean difference, SOFA: Sequential Organ Failure Assessment, SL: support level

**Supplementary Table 6.** Time-to-event analysis using a cause-specific Cox proportional hazards model for the risk factors for early (≤2 days) and all ICU readmissions

|  | Non-readmission |  |  | Non-readmission |  |
| --- | --- | --- | --- | --- | --- |
|  | vs. early |  |  | vs. all |  |
|  | readmission |  |  | readmission |  |
|  | Hazard ratios | P |  | Hazard ratios | P |
| Characteristics | (95% CIs) | value |  | (95% CIs) | value |
| **Patient characteristics** |  |  |  |  |  |
| Age, years | 1.00 (1.00–1.00) | 0.465 |  | 1.00 (1.00–1.00) | 0.551 |
| Male | 1.09 (1.02–1.17) | 0.013 |  | 1.19 (1.15–1.23) | 0.000 |
| Charlson Comorbidity Index | 1.05 (1.02–1.08) | 0.000 |  | 1.04 (1.03–1.05) | 0.000 |
| Long-term care needs, n (%) |  |  |  |  |  |
| No care needs | Ref. | – |  | Ref. | – |
| SL1-2 and CNL1 | 1.12 (0.98–1.28) | 0.088 |  | 1.17 (1.09–1.25) | 0.000 |
| CNL 2-5 | 1.02 (0.88–1.18) | 0.816 |  | 0.99 (0.92–1.07) | 0.870 |
| Admission classification |  |  |  |  |  |
| Elective surgery | Ref. | – |  | Ref. | – |
| Emergency surgery | 1.94 (1.69–2.22) | 0.000 |  | 2.04 (1.91–2.17) | 0.000 |
| Non-surgery | 1.28 (1.08–1.52) | 0.004 |  | 0.98 (0.91–1.04) | 0.474 |
| Primary diagnosis category |  |  |  |  |  |
| Cancer | 1.01 (0.76–1.34) | 0.945 |  | 0.98 (0.90–1.07) | 0.646 |
| Acute coronary syndrome | 1.07 (0.93–1.22) | 0.357 |  | 1.16 (1.08–1.24) | 0.000 |
| Aortic dissection or aneurysm | 1.30 (1.16–1.47) | 0.000 |  | 1.51 (1.40–1.62) | 0.000 |
| Stroke | 0.99 (0.83–1.18) | 0.923 |  | 0.93 (0.85–1.02) | 0.130 |
| Acute abdominal diseases | 1.24 (1.07–1.43) | 0.003 |  | 1.10 (1.03–1.18) | 0.004 |
| Acute heart failure | 1.22 (1.03–1.44) | 0.022 |  | 1.45 (1.34–1.57) | 0.000 |
| Sepsis | 1.13 (0.94–1.36) | 0.194 |  | 1.19 (1.10–1.28) | 0.000 |
| Trauma | 1.22 (1.03–1.45) | 0.019 |  | 0.﻿98 (0.88–1.08) | 0.630 |
| Total SOFA score at ICU admission | 1.02 (0.98–1.06) | 0.313 |  | 0.﻿﻿99 (0.98–1.00) | 0.192 |
| Total SOFA score at ICU discharge | 1.07 (1.03–1.12) | 0.002 |  | 1.05 (1.04–1.07) | 0.000 |
| SOFA subscore ≥2 at ICU discharge |  |  |  |  |  |
| Respiratory | 1.30 (1.20–1.41) | 0.000 |  | 1.05 (1.00–1.11) | 0.037 |
| Platelet | 1.03 (0.95–1.12) | 0.451 |  | 1.01 (0.96–1.06) | 0.811 |
| Liver | 0.99 (0.86–1.14) | 0.883 |  | 1.09 (1.03–1.15) | 0.002 |
| Circulatory | 1.53 (0.92–2.53) | 0.102 |  | 1.12 (0.94–1.33) | 0.218 |
| Neurological | 0.94 (0.81–1.09) | 0.399 |  | 0.82 (0.77–0.87) | 0.000 |
| Renal | 1.14 (1.01–1.30) | 0.041 |  | 1.24 (1.17–1.31) | 0.000 |
| Length of first ICU stay |  |  |  |  |  |
| 1 day | Ref. | – |  | Ref. | – |
| 2 days | 1.02 (0.76–1.38) | 0.876 |  | 0.77 (0.68–0.87) | 0.000 |
| 3–6 days | 1.16 (0.86–1.56) | 0.345 |  | 0.93 (0.83–1.06) | 0.277 |
| ≥7 days | 1.22 (0.89–1.66) | 0.216 |  | 1.03 (0.91–1.17) | 0.674 |
| Step-down transfer from ICU to IMCU | 0.80 (0.65–0.95) | 0.016 |  | 0.92 (﻿﻿0.76–1.05) | 0.140 |
| Fiscal year |  |  |  |  |  |
| 2018–2019 | Ref. | – |  | Ref. | – |
| 2020–2022 | 1.08 (0.91–1.28) | 0.365 |  | 1.03 (﻿0.95–1.11) | 0.503 |
| Season |  |  |  |  |  |
| Spring | Ref. | – |  | Ref. | – |
| Summer | 1.11 (1.02–1.22) | 0.019 |  | 1.03 (0.99–1.07) | 0.164 |
| Fall | 1.11 (1.02–1.21) | 0.013 |  | 1.04 (1.00–1.08) | 0.053 |
| Winter | 0.99 (0.90–1.08) | 0.755 |  | 0.95 (0.91–0.99) | 0.011 |
| Weekend discharge from ICU | 0.93 (0.84–1.02) | 0.126 |  | 0.93 (0.89–0.96) | 0.000 |
| **Hospital characteristics** |  |  |  |  |  |
| Reimbursed code of ICU |  |  |  |  |  |
| A3011 | Ref. | – |  | Ref. | – |
| A3012 | 0.81 (0.55–1.20) | 0.289 |  | 0.98 (0.84–1.16) | 0.851 |
| A3013 | 0.75 (0.61–0.92) | 0.006 |  | 0.84 (0.﻿75–0.95) | 0.005 |
| A3014 | 0.66 (0.48–0.92) | 0.014 |  | 0.81 (0.﻿﻿68–0.98) | 0.031 |
| Number of ICU beds | 1.01 (0.99–1.03) | 0.322 |  | 1.01 (1.00–1.02) | 0.010 |
| Hospital with IMCU beds | 0.90 (0.71–1.15) | 0.406 |  | 0.98 (0.85–1.14) | 0.798 |
| Total number of hospital beds | 1.00 (1.00–1.00) | 0.483 |  | 1.00 (1.00–1.00) | 0.893 |
| Academic hospital | 1.15 (0.96–1.38) | 0.137 |  | 1.07 (0.93–1.24) | 0.333 |
| Annual volume of ambulance acceptance | 1.00 (1.00–1.00) | 0.434 |  | 1.00 (1.00–1.00) | 0.994 |
| Tertiary emergency hospital | 0.97 (0.78–1.21) | 0.815 |  | 0.87 (0.77–0.99) | 0.033 |

CNL: care-needs level, CI: confidence interval, HR: hazard ratio, ICU: intensive care unit, IMCU: intermediate care unit, IMV: invasive mechanical ventilation, OR: odds ratio, RRT: renal replacement therapy, SOFA: Sequential Organ Failure Assessment, SL: support level
Cause-specific Cox regression was performed by treating in-hospital death as a censoring event, including patients who died in-hospital without ICU readmission (previously excluded from the main analysis). Cluster-robust standard errors were applied at the hospital level.
